# Accurate Multiple Ocean Bottom Seismometer Positioning in Shallow Water Using GNSS/Acoustic Technique

**DOI:** 10.3390/s19061406

**Published:** 2019-03-21

**Authors:** Huimin Liu, Zhenjie Wang, Shuang Zhao, Kaifei He

**Affiliations:** 1 School of Geosciences, China University of Petroleum (East China), Qingdao 266580, China; zsunique.vip@gmail.com (S.Z.); hekaifei983@163.com (K.H.); 2 Laboratory for Marine Mineral Resources, Qingdao National Laboratory for Marine Science and Technology, Qingdao 266071, China

**Keywords:** GNSS/Acoustic technique, ocean bottom seismometers, sound ray tracing, sound ray bending, incidence angle model

## Abstract

The Global Navigation Satellite System combined with acoustic technique has achieved great economic benefits in positioning of ocean bottom seismometers, with hundreds of underwater transponders attached to seismometers typically being deployed during oil exploration. The previous single transponder positioning method ignored the similar underwater environments between the transponders. Due to the refraction effect of sound, the technique usually showed poor positioning accuracy in shallow water when the incidence angles are large. In this paper, the effect of sound ray bending is analyzed based on the sound ray tracing method in shallow water, and a new piecewise incidence angle model is proposed to improve the positioning accuracy of multiple objects in order to estimate the sound ray bending correction. The parameters of the new model are divided into groups and estimated by sequential least squares method, together with all of the transponders. The observability analysis is discussed in simulation and testing experiments in the South China Sea. The results show that the newly proposed method is able to make full use of the acoustic observation data of hundreds of transponders to accurately estimate the SRB correction, which could also significantly improve the positioning accuracy of multiple transponders.

## 1. Introduction

During data acquisition using ocean bottom seismometers (OBS) in shallow water, it is usually necessary to locate a large number of submarine seismometers [1]. There are two traditional methods for locating the position of seismometers, one of which is the first break secondary positioning (FBSP) method [2,3]. The positioning accuracy of this method is generally meters (3~10 m), and higher positioning precision for a large number of seismometers within the sub-meter level is a challenge. The other method combines the Global Navigation Satellite System (GNSS) and the acoustic ranging technique, and provides important positioning measurements for underwater objects [4,5,6,7,8]. This method is able to utilize low-cost underwater transponders, which are small and lightweight, and are easily attached to the seismic cable or OBS. This technique can get the position of OBS more accurately than FBSP in shallow water, and has been proved to satisfy the requirements of oil exploration [6,7].

The Real-Time Kinematic (RTK) GNSS technique is always employed for GNSS/acoustic surveys, which achieve a 2–3 cm positioning accuracy and provide a stable absolute position reference [9]. However, the position of exploration vessel is usually far from land for oil exploration and can only be estimated with an accuracy at the decimeter level utilizing Global Navigation Satellite System (DGNSS) technology (e.g., StarFire, VeriPos and Marinestar) based on the communication data link of Inmarsat [6,7]. In contrast to the electromagnetic ranging technique, the acoustic range is the product of sound speed and travel time in the case of ignoring the sound refraction. As a development of the underwater acoustic ranging technique, time delay errors caused by system hardware are effectively controlled and eliminated. Although the speed of sound in the ocean can be directly measured by a conductivity temperature depth gauge (CTD) or sound speed profiler (SSP) gauge, the errors induced by the inaccuracy of SSP and sound refraction are the main factors affecting underwater acoustic ranging. The acoustic ranging is directly affected by the variation of sound speed along the trajectory [10,11]. When the incidence angle is greater than 85 degrees, the bending error of the sound line can reach more than 0.15 m in 100 m of water.

Quite a few studies have measured and analyzed SSP with different techniques to reduce the errors caused by sound speed and sound ray bending (SRB) [12,13,14,15,16,17,18,19,20,21,22,23,24,25]. Previous methods, such as the epoch difference method [12] and the time model [15,17], are usually used under conditions with long observation times, where the SRB changes more significantly over time, and are not convenient during shallow water oil exploration. During OBS acoustic positioning, all the transponders only obtain a small amount of the observed data, and SRB with a high incidence angle has a great effect on positioning. The effect of an inaccurate stochastic model on the underwater positioning accuracy in shallow water has been analyzed [21], and the incidence angle segmented cosine form was used to improve positioning accuracy. This was an empirical approach, and did not analyze the function relation between the SRB and the incidence angle in detail. From the perspective of position accuracy and scientific research, it is more meaningful to explore the relationship between acoustic bending and incidence angle.

Therefore, the inaccurate sound speed and the SRB become the major factors influencing the accuracy of acoustic OBS positioning. The traditional estimated model without sound speed is usually adopted when the sound speed is inaccurate [17]. However, the OBS acoustic data of single transponders are not enough to correct for inaccurate sound speed. As hundreds of transponders hang on an underwater cable, they can provide an abundance of acoustic observations to model the acoustic refraction and estimate the SRB correction. In this research, the Ocean Bottom Cable (OBC) positioning system is introduced, and a relevant method for positioning single transponder is given. Then we analyze the characteristics of the SRB in shallow water based on the sound ray tracing (SRT) method. With the above analysis, we present a new segment incidence angle (SIA) model in shallow water. In the new method, the SRB correction can be divided into groups based on the incidence angles, and the position parameters and model parameters can be estimated together using the Sequence Least Square (SLS) method. Simulation and testing experiments in the South China Sea are used to verify and evaluate the new method.

This paper is organized as follows. Section 2 introduces the OBC measurement system in shallow water and provides details about the estimation method for survey vessel position and transponders. The SRT method, SRB correction and mathematical formulations used for the estimation of each position of seafloor transponders will be described in Section 3. This section also presents a new SIA model, and describes the new approach to positioning multiple transponders. Section 4 introduces the simulation and experiments. Finally, Section 5 presents the conclusions of the study.

## 2. Ocean Bottom Cable Measurement System and GNSS/Acoustic Technique

### 2.1. Ocean Bottom Cable Measurement System

**Figure 1:**
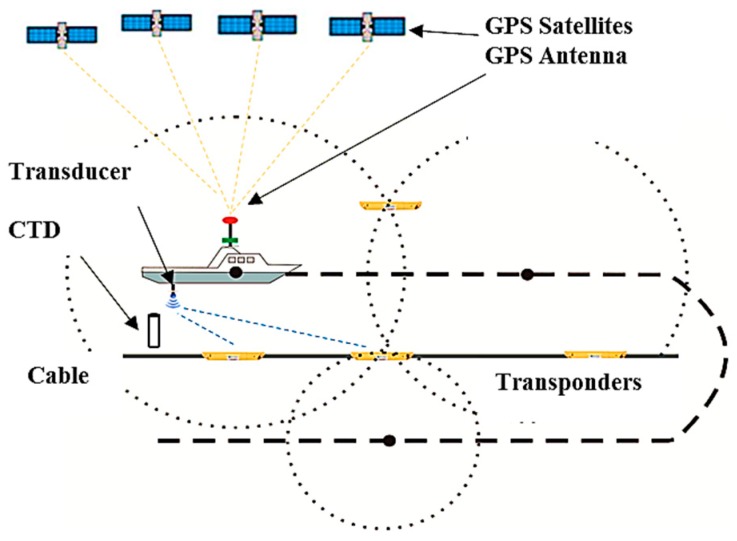
Schematic of the GNSS/acoustic ocean bottom cable positioning system.

Figure 1 illustrates the ocean bottom cable positioning system for shallow water oil exploration. This system includes a sea floor cable with hundreds of transponders attached, a deck unit with a GNSS antenna, and a dunking transducer on a rigid pole. The global differential GNSS technology can be utilized far away from the mainland, and the level of positioning accuracy (Veripos) is better than 30 cm [26]. The relative displacement between the GNSS antennas and the transducer can be calculated using attitude angles. The survey ship sails along the survey line after the cable has been deployed, and the transducer will interrogate the sea floor transponders. A deck unit commands the transducer, which transmits and receives acoustic signals and measures the travel times from the transducer to the seafloor acoustic transponders. The number of acoustic observations for a transponder will typically be less than one hundred, and some of them have large incidence angles.

The SSP can be obtained by SSP gauge or CTD, and the mean speed can be calculated using the weighted or equivalent SSP method [22,26]. The sampling rate of kinematic GNSS is generally 1Hz, and the acoustic system is usually near 10 s, so the position of GNSS at the acoustic sampling time is usually obtained through the Lagrange interpolation method. The survey vessel surveys in a circle in the process of exploration and positioning to reduce the ray bending error with parallel and symmetrical observation structures. The acoustic transmission source level is 185 dB and the receiver sensitivity is 110 dB. This can be well applied in the acoustic environment of shallow water, and the ranging accuracy is better than 0.5 m at a range of 100 m [3,4,5,6].

### 2.2. The Positioning of the Survey Vessel

The relationship of coordinates between the GNSS antenna and the reference point can be expressed by [5]:

(1) $$\left[\begin{array}{c} x\\ \begin{array}{c} y\\ z \end{array} \end{array}\right]=\left[\begin{array}{c} x_{gps}\\ y_{gps}\\ z_{gps} \end{array}\right]R(h)R(p)R(r)\left[\begin{array}{c} \Delta x_{1}\\ \Delta y_{1}\\ \Delta z_{1} \end{array}\right]$$

(2) $$R(h)=\left[\begin{array}{ccc} \cos h & \sin h & 0\\ -\sin h & \cos h & 0\\ 0 & 0 & 1 \end{array}\right],R(p)=\left[\begin{array}{ccc} \cos p & 0 & \sin p\\ 0 & 1 & 0\\ -\sin p & 0 & \cos p \end{array}\right],R(r)=\left[\begin{array}{ccc} 1 & 0 & 0\\ 0 & \cos r & \sin r\\ 0 & -\sin r & \cos r \end{array}\right]$$

where $\left(x_{gps},y_{gps},z_{gps}\right)$ represents the coordinates of the GNSS antenna, and h, p and r represent the attitude measurements of heading, pitch and rolling angles, respectively. This can be measured by an electrical gyrocompass and an attitude sensor, or by GNSS attitude determination. $\left(\Delta x_{1},\Delta y_{1},\Delta z_{1}\right)$ represents the baseline between the GNSS antenna and the reference point, which is defined as the origin of the body-fixed coordinate frame. (x,y,z) represents the coordinates of the reference point.

The position of transponders can also be computed using the following formulation:

(3) $${\left[\begin{array}{c} x\\ \begin{array}{c} y\\ z \end{array} \end{array}\right]}_{T}=R(h)R(p)R(r){\left[\begin{array}{c} x\\ \begin{array}{c} y\\ z \end{array} \end{array}\right]}_{T_{0}}$$

where ${\left(x,y,z\right)}_{T}$ denotes the coordinates of the transducer in the navigation coordinate system with the transformation of ship attitude, and ${\left(x,y,z\right)}_{T_{0}}$ represents the original coordinates of the transducer in the ship coordinate system and can be directly measured by a total station instrument while the ship is in dry-dock. The main function of the total station instrument is to survey the relative coordinates.

### 2.3. Calculation of Transponder Positions

Generally, the time delay can be measured and corrected by the instrument manufacturer, and the observation equation can be expressed as [12]

(4) $$\rho (i,k)=f\left(X(i,k),X_{T}^{}(k)\right)+\delta \rho_{v}(i,k)+\varepsilon (i,k)$$

(5) $$f(X_{T}^{}(k),X(i,k))=\sqrt{{\left(x_{T}^{}(k)-x(i,k)\right)}^{2}+{\left(y_{T}^{}(k)-y(i,k)\right)}^{2}+{\left(z_{T}^{}(k)-z(i,k)\right)}^{2}}$$

where ρ(i,k) represents the acoustic ranging at time i. k represents the transponder mark, and $f(X_{T}^{}(k),X(i,k))$ represents the geometric distance between the transducer and the transponder. X and $X_{T}^{}$ represent the coordinates of the transducer and the transponder, respectively. $\delta \rho_{v}(i,k)$ represents the systematic error due to the refraction of sound rays, and ε(i,k) represents the Gaussian noise. 

The mean sound velocity (MSV) can be measured and calculated, or determined based on experience, and the linearized observation equation is as follows:

(6) $$\rho_{o}^{}(i,k)-f\left(X(i,k)-X_{T}^{}(k)\right)=a_{o}^{}(i,k)dx+\delta \rho_{v}(i,k)+\varepsilon (i,k)+b_{o}^{}(i,k)\varepsilon_{p}(i,k)$$

where $\rho_{o}^{}(i,k)=C_{e}\cdot t(i,k)$, $C_{e}$ represents the MSV, and t(i,k) represents the travel time; $a_{o}^{}(i,k)=\left[\begin{array}{ccc} \frac{\partial f_{i}^{k}}{\partial x} & \frac{\partial f_{i}^{k}}{\partial y} & \frac{\partial f_{i}^{k}}{\partial z} \end{array}\right]$ is the Jacobian matrix of the measurement equation, and $\frac{\partial f_{i}^{k}}{\partial x}=\frac{x_{T_{o}}^{}(k)-x(i,k)}{f_{o}(i,k)}$, $\frac{\partial f_{i}^{k}}{\partial y}=\frac{y_{T_{o}}^{}(k)-y(i,k)}{f_{o}(i,k)}$, $\frac{\partial f_{i}^{k}}{\partial z}=\frac{z_{T_{o}}^{}(k)-z(i,k)}{f_{o}(i,k)}$, $f_{o}(i,k)=\sqrt{{\left(x_{T_{o}}^{}(k)-x(i,k)\right)}^{2}+{\left(y_{T_{o}}^{}(k)-y(i,k)\right)}^{2}+{\left(z_{T_{o}}^{}(k)-z(i,k)\right)}^{2}}$. $b_{o}^{}(i,k)$ represents the first partial derivatives with respect to X, and $\varepsilon_{p}$ represents the GNSS antenna error at the position X. If the impact of sound speed variation is small, the $\delta \rho_{v}$ can be ignored. dx represents the position correction of the transponder. The position correction can be estimated by the least squares method. This estimation method will subsequently be represented by LS1 in the experiment.

In shallow water, the SRB changes sharply with the incidence angle for one transponder. When the incidence angle is greater than 65 degrees, the SRB curvature is obvious. In practice, the observation data are selected according to the cut incidence angle, which can generally reduce the influence of the bending error. This method will later be represented by LS2 in the experiment.

If the SSP is not measured, we can give an initial value of sound speed and estimate its correction. The observation equation can be expressed as [17]

(7) $${\tilde{\rho }}_{o}(i,k)-f\left(X(i,k)-X_{To}^{}(i)\right)=a_{o}(i,k)dx+t(i,k)dc+\delta \rho_{v}(i,k)++\varepsilon (i,k)+b_{o}(i,k)\varepsilon_{p}(i,k)$$

where ${\tilde{\rho }}_{o}(i,k)=C_{o}^{}\cdot t(i,k)$, and $C_{o}^{}$ can be set to 1500 m/s. This method is usually used in the absence of correct MSV, and it will later be represented by LS3 in the experiment.

## 3. New Approach to Positioning Multiple Transponders

### 3.1. The Effect of SRB in Shallow Water

**Figure 2:**
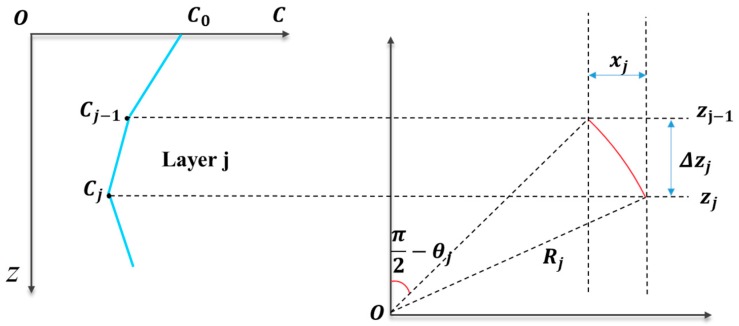
Layering gradient and sound ray tracing.

The SRT method with constant gradient is introduced in this section. If the sound speed changes little or uniformly, then we can consider the sound speed gradient to be a constant. As shown in Figure 2, $C_{j}$ represents the sound speed in water layer j, and $z_{j}$ represents the depth of the water. The travel of sound in water follows Snell’s law [10,11]

(8) $$\sin\theta_{j}/C_{j}=P$$

where $\theta_{j}$ represents incidence angle, and P is a constant. In each layer, we assume that the medium changes uniformly. In other words, the sound speed has a constant gradient, and the track of the ray is a continuous arc. The radius $R_{j}$ of the arc can be expressed as [27]:

(9) $$R_{j}=-1/Pg_{j}$$

where $g_{i}$ represents the sound speed gradient. The horizontal distance of the sound ray $x_{j}$ can be computed as follows

(10) $$x_{j}=\frac{{\left[1-{\left(PC_{j-1}\right)}^{2}\right]}^{1/2}-{\left[1-P^{2}\left(C_{j-1}+g_{j}\Delta z_{j}\right)\right]}^{1/2}}{Pg_{j}}$$

where $\Delta z_{j}$ represents the length of layer j. In general, the interval between layers is wide if the variation of sound velocity between layers is small. The length of arc $S_{j}$ in layer j can be computed by [28]

(11) $$S_{j}=R_{j}\left(\theta_{j-1}-\theta_{j}\right)$$

Thus, the travel time is estimated by

(12) $$t_{j}=\frac{\theta_{j-1}-\theta_{j}}{Pg_{j}^{2}}\ln\left[\frac{C_{j}}{C_{j-1}}\right]=\frac{\arcsin\left[P\left(C_{j-1}+g_{j}\Delta z_{j}\right)\right]-\arcsin\left(PC_{j-1}\right)}{Pg_{j}^{2}}\ln\left[1+\frac{g_{j}\Delta z_{j}}{C_{j-1}}\right]$$

In OBS underwater acoustic positioning, a fixed weighted mean sound velocity (WMSV) is usually used in the calculation, and the WMSV can be calculated by [29]

(13) $$C_{w}=\frac{1}{H}\sum_{j=1}^{N}w_{j}\frac{\left(C_{j-1}+C_{j}\right)\left(\Delta z_{j}\right)}{2}$$

where H represents the depth of water; $w_{j}$ is the weight of each water column related to the incidence angle, sound speed structure and other factors. If $w_{j}=1$, the $C_{w}$ represents the MSV.

The MSV assumes a single sound speed value for the entire localization area. In fact, the calculated MSV is different due to the incidence angles and sound ray, and can cause the SRB error. The SRB correction $\delta \rho_{v}$ can be modeled and absorbed by the MSV in many forms, such as a constant, 1st- or 2nd-degree polynomial with a time series [15] as

(14) $$C_{e}=a_{0},C_{e}=a_{0}+a_{1}(t_{i}-t_{0})\;\mathrm{or}\;C_{e}=a_{0}+a_{1}(t_{i}-t_{0})+a_{2}{\left(t_{i}-t_{0}\right)}^{2}$$

The SRB correction $\delta \rho_{v_{}}$ can also be modeled directly [17] as

(15) $$\delta \rho_{v_{}}(i)=a\left(t_{i}-t_{ma}\cdot \cos\lambda_{ma}\right)$$

where a is the coefficient to be estimated, and $t_{i}$ represents the travel time. $t_{ma}$ represents the minimum travel time among the measured epochs. $\lambda_{ma}$ represents the incidence angle according to the calculation of the position of the transducer. 

The above methods are usually used under conditions where there is a long observation time, and the SRB varies with time more significantly. Based on the above methods, we can analyze the SRB in shallow water, which helps us to find some useful models to describe it quantitatively.

**Figure 3:**
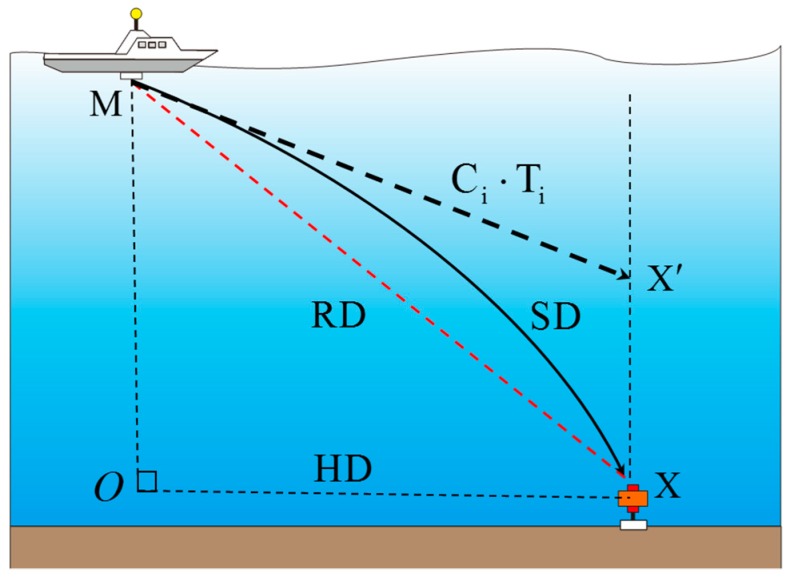
The schematic of the SRB.

The relationship among the horizontal distance (HD), geometrical distance (RD) and sound ray trace (SD) is shown in Figure 3. According to the analysis of the above, the acoustic ranging is usually treated as a straight line, while the real sound track is a curve in inhomogeneous water. In this research, we ignore the complexity of the top surface sound speed, and consider the analysis of the steady acoustic environment in small areas within a short period, as the OBS measurement area is only a few hundred square meters, and a single operation period is not usually more than an hour. We temporarily ignore the complexity of those short sound speed changes, and combine all the observation data to estimate the SRB error in this region. We only consider the commonness between these observation data; for example, under the same incidence, the SIB error is approximately equivalent. We use these characteristics and combine all the observation data to estimate the bending error of the acoustic line in this region.

Based on the above equation, the SRB can be simulated using the measured SSP. Assuming that the coordinates of the transponder and the transducer are known, the beam incidence angle and travel time, as unknown parameters, can be solved by Newton iteration or other secant methods using the SSP.

**Figure 4:**
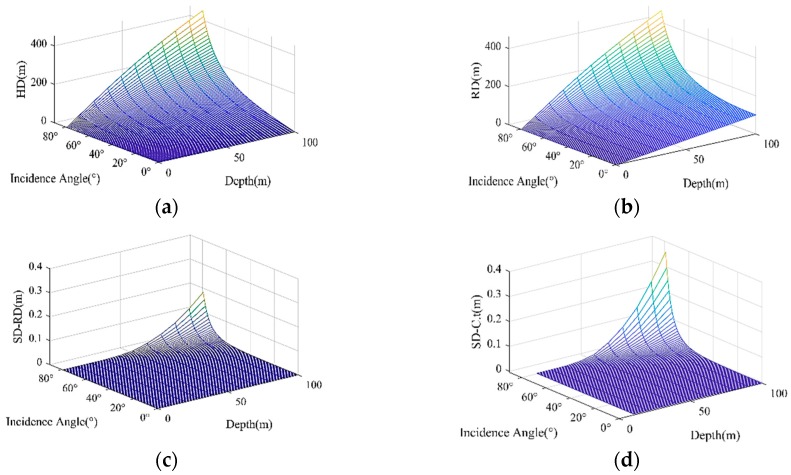
The range with different incidence angles at a depth of 10~100 m. (**a**) Horizontal distance (HD) with the change of incidence angle and depth; (**b**) geometric distance (RD) with the change of incidence angle and depth; (**c**) the difference between sound ray trace (SD) and RD; (**d**) the difference between sound ray trace and C⋅t, where the C represents the MSP.

As shown in Figure 4, we can get some information from the above figures. The HD, RD, SD−RD and SD−C⋅t increase significantly with the increase of incidence angle and water depth, and show little change when the incidence angle is smaller than 60 degrees in shallow water. The effect of SRB can reach 0.18 m at a depth of 100 m. The largest HD can reach 450 m when the incidence angle is 80 degrees, and this determines the measured horizontal range.

From the above analysis (Section 3.1), the SRB correction can be modeled approximately according to incidence angle at the same depth.

### 3.2. A Segmented Incidence Angle (SIA) Model

In this research, we assume that the SSP is inaccurate or the CTD has not been calibrated; thereby, the MSV and the SRB become the major factors affecting the accuracy of the acoustic positioning. This problem is also the most common for processing acoustic ranging data. In order to build an accurate fitting model, the parameters of SRB correction are divided into different groups by the incidence angles, and we can find a model through many simulation experiments. A new model is proposed to estimate the SRB correction according to the incidence angles

(16) $$\delta \rho_{v}(i)=w_{M}/((\cos(\theta_{i}){)}^{N}+b_{M}\begin{array}{cc} \begin{array}{ccc} & N=3 & or \end{array} & 4 \end{array}$$

where $\theta_{i}$ represents the incidence angle, and $w_{M}$ represents the coefficient of the model. $b_{M}$ represents the constant deviation due to inaccurate MSV. N represents the cosine of the incidence angle to the N. When the incidence angle is less than 60°, the SRB correction can be neglected according to the analysis in the previous section.

**Figure 5:**
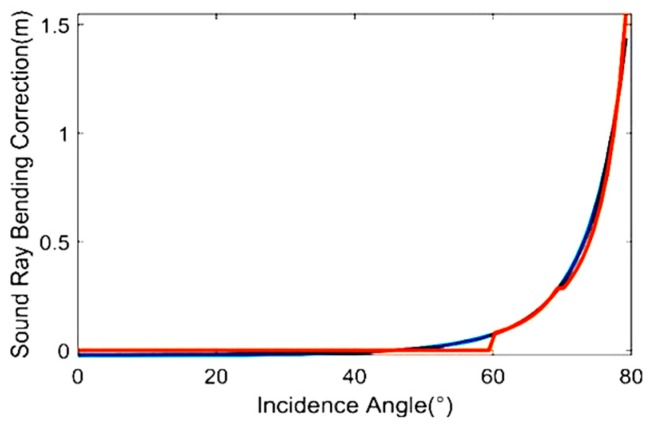
The SRB correction using the SIA model.

As shown in Figure 5, the new SIA model can fit well with the SRB correction by selecting the parameters. The new model is used to fit the SRB correction, and N is selected as 3 or 4, corresponding to segments 60–70° or 70–80°, depending on the rising trend.

In shallow water OBS acoustic positioning, the area of multiple transponder positioning is normally small (one square kilometer). All transponders are located on the shallow and flat seabed (most shallow sea exploration terrain meets this condition); then, the water depth of the area can even be used as a fixed value. Assuming that the effect on SSP caused by the non-barotropic tidal flow or internal gravitational wave in a short time is small, the observation environment will be similar for all transponders within a short time. There are reasons to believe that the same incidence angles of acoustic ranging have the same SRB, which increases with the increase of the incidence angle. Naturally, the key issue is to select correct parameters, and the next section focuses on this problem. A high-precision underwater positioning algorithm for multiple targets with regard to acoustic line bending error is presented.

### 3.3. Calculation Method of Multiple Transponders with Sequential Least Square

For a single transponder, it is easy to cause ill-posed problems due to the introduction of many model parameters. From the above analysis, if the observations of different transponders have the same incidence angle, the same SRB correction can be estimated together. A large number of observations will place a huge burden on the computer. Based on the Sequential Least Square (SLS) method and the matrix orthogonal principle, a convenient solution is given in this research.

We first use the LS1 method or the LS2 method to calculate the initial transponder coordinates, and the observed values can be categorized according to the threshold values and incidence angles. Then, the unknown parameters $dX_{T}^{}(k)=\left[dx_{T}^{}(k),dy_{T}(k),dz_{T}^{}(k),\underset{dc}{\underbrace{w_{1}^{},\ldots ,w_{m}^{}}}\right]$, and the new observation equation can be expressed as

(17) $$L_{a}(k)=B_{a}(k)dX_{T}^{}(k)+\Delta (k)$$

where Δ represents the sum of random errors and modeling errors, and $B_{a}=\left[B_{1},B_{2}\right]$. $L_{a}$ represents the linearized observation.

(18) $$\begin{array}{c} B_{1}(k)={\left[\begin{array}{c} a_{o}(1,k)\\ a_{o}(2,k)\\ \vdots \\ a_{o}(n,k) \end{array}\right]}_{n\times 3}\\ B_{2}(k)={\left[\begin{array}{cccc} 0_{n_{2}\times 1} & 0_{n_{2}\times 1} & 0_{n_{2}\times 1} & 0_{n_{2}\times 1}\\ 1/((\cos(\theta (1,k)){)}^{N} & 0 & 0 & 0\\ 0 & 1/((\cos(\theta (2,k)){)}^{N} & 0 & 0\\ 0 & 0 & \ddots & 0\\ 0 & 0 & 0 & 1/((\cos(\theta (m,k)){)}^{N} \end{array}\right]}_{n\times m} \end{array}$$

(19) $$L_{a}(k)=\left[\begin{array}{c} {\tilde{\rho }}_{o}(1,k)-f\left(X(1,k)-X_{To}^{k}\right)\\ {\tilde{\rho }}_{o}^{}(2,k)-f\left(X(2,k)-X_{To}^{k}\right)\\ \vdots \\ {\tilde{\rho }}_{o}^{}(n,k)-f\left(X(n,k)-X_{To}^{k}\right) \end{array}\right]$$

and $B_{1}$ represents the Jacobian matrix after linearization. $B_{2}$ represents the coefficients of sound correction, and $p_{1}+p_{2}+\cdots +p_{m}=n_{1}=n-n_{2}$. m represents the number of groups. $n_{1}$ is the number of observations where the incidence angle is larger than 65 degrees, and n is the number of all transponder observations. According to the matrix orthogonal principle, the simplification of (17) is as follows

(20) $$R_{B1}B_{2}dc=R_{B_{1}}L_{a}$$

where $R_{B_{1}}=I-B_{1}{\left(B_{1}^{T}\sum_{l}^{-1}B_{1}\right)}^{-1}B_{1}^{T}\sum_{l}^{-1}$, and I represents an identity matrix. $\sum_{l}^{}$ represents the covariance of observation $L_{a}$, and dc represents the SIA parameters.

Then the SLS method can be used to estimate the SIA parameters [30].

(21) $$\left\{ \begin{array}{l} {\hat{X}}_{1}=P_{{\hat{X}}_{1}}^{-1}A_{1}^{T}L_{1}\\ {\hat{X}}_{2}=P_{{\hat{X}}_{2}}^{-1}\left(A_{2}^{T}P_{2}L_{2}+P_{{\hat{X}}_{1}}^{-1}{\hat{X}}_{1}\right)\\ \vdots \\ {\hat{X}}_{k}=P_{{\hat{X}}_{k}}^{-1}\left(A_{k}^{T}P_{k}L_{k}+P_{{\hat{X}}_{k-1}}^{-1}{\hat{X}}_{k-1}\right) \end{array}\right.$$

(22) $$\left\{ \begin{array}{l} P_{{\hat{X}}_{1}}=A_{1}^{T}P_{1}A_{1}\\ P_{{\hat{X}}_{2}}=P_{{\hat{X}}_{1}}+A_{2}^{T}P_{2}A_{2}\\ \vdots \\ P_{{\hat{X}}_{k}}=P_{{\hat{X}}_{k-1}}+A_{k}^{T}P_{k}A_{k} \end{array}\right.$$

where k represents the transponder ID, and $A_{k}={\left(R_{G1}B_{2}\right)}_{k}$. ${\hat{X}}_{k}=\hat{d}c$, and its variance-covariance matrix can be expressed by

(23) $${\hat{\sigma }}_{0_{k}}^{2}=\frac{V_{k}^{T}P_{k}V_{k}+{\left({\hat{X}}_{k}-{\hat{X}}_{k-1}\right)}^{T}P_{{\hat{X}}_{k-1}}\left({\hat{X}}_{k}-{\hat{X}}_{k-1}\right)}{n_{k}}$$

(24) $$\Sigma_{{\hat{X}}_{k}}=Q_{{\hat{X}}_{k}}{\hat{\sigma }}_{0_{k}}^{2}=P_{{\hat{X}}_{k}}^{-1}{\hat{\sigma }}_{0_{k}}^{2}$$

**Figure 6:**
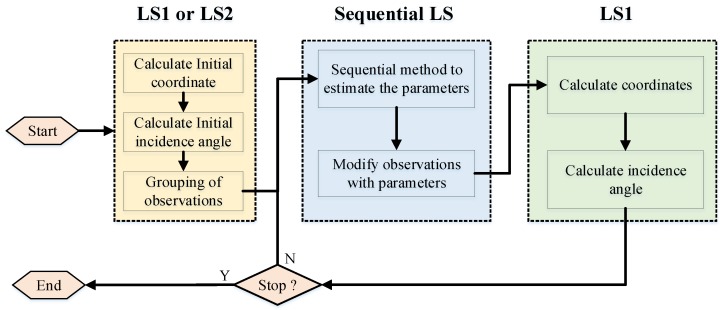
Flowchart of calculation method of multiple transponders using the SLS method.

As shown in Figure 6, for the final transponder, SIA parameters and variance-covariance matrix can be calculated by solving Equations (21) and (22). When the final new model parameters of the SRB correction are solved, they are brought into Equation (17). Thus, the coordinates and incidence angles can be estimated at this time using the LS1 method again. The new results are brought into the sequential least squares algorithm and iterated until there are few differences from the previous results. 

If the effect on SSP caused by the non-barotropic tidal flow or internal gravitational wave in a short time cannot be ignored, the new model parameters can be remodeled and estimated for sections of time. Only some new modeling parameters are added and estimated here. Certainly, we could also use the other models (see Equation (14) or Equation (15)) to reduce the impact of this change on positioning. Real-time and high-precision SSP is difficult to obtain in OBC positioning, and it is usually measured before or after positioning. The method proposed in this research is another way of improving its accuracy based on estimating the parameters of the acoustic bending model.

## 4. Simulation and Experiment in the South China Sea

### 4.1. Case 1: Simulation

**Figure 7:**
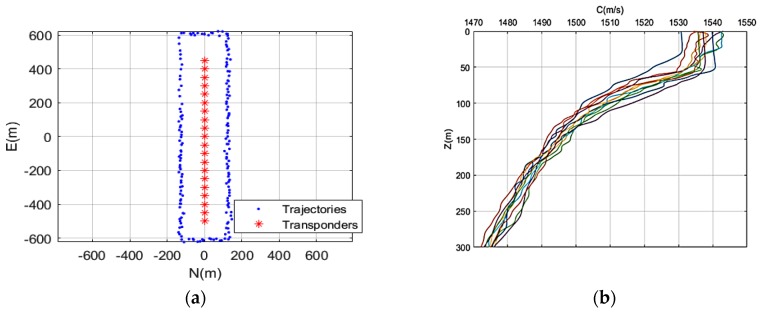
The surveying trajectories (depth = 300 m) and sound speed profiles. (**a**) The blue points are the surveying trajectories, and the red asterisks are the transponders on the seafloor; (**b**) Sound speed profiles of water column derived from 12 months.

This experiment is designed to verify the accuracy attainable using the new methods for multiple transponders. As shown in Figure 7a, 20 transponders are simulated at depths of 100 m, 200 m and 300 m, and their horizontal distances shrunk from the surveying trajectories are 150 m, 250 m and 350 m. As shown in Figure 7b, since the environments are similar within a short period (for example, 1 h), we will obtain the round-trip times using Snell’s law of refraction with 12 months’ SSP of the same region. The positioning errors of the transducers are 10 cm in the horizontal direction and 30 cm in the vertical direction, and the random errors in travel time are 10 µs. The incidence angles range from 40° to 80°, which correspond to the underwater acoustic data in the process of offshore oil exploration. The speed of the ship is 3 knots, and the sampling period of the transducers are 10 s and 45 s, which correspond to good and bad observation conditions. At the same time, in order to approximate the actual situation, the distribution of the transponder was arranged in the form of cosine curve in the bad case to simulate the asymmetry of observation.

**Figure 8:**
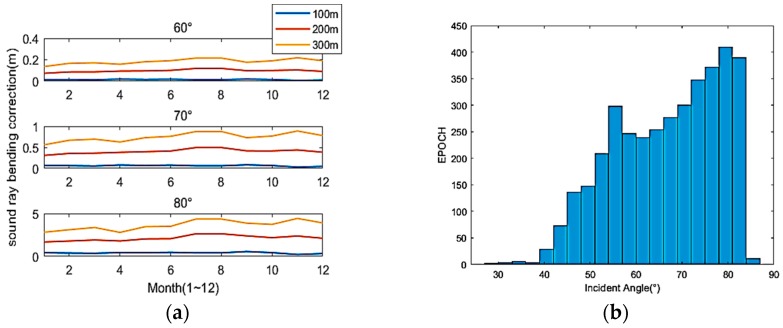
The SRB correction during different months, and the histogram of the incidence angles (depth = 300 m). (**a**) The horizontal axis marks the months from January to December, and incidence angles of 60°, 70° and 80°; (**b**) The horizontal axis marks the incidence angles, and epoch is the total number of acoustic data.

As shown in Figure 8a, the SRB correction is related to the incidence angles, depth and time, and increases sharply with the increase of incidence angle and depth. From the histogram of the incidence angle as shown in Figure 8b, we can find that there are many incidence angles greater than 65°, and the problem of SRB must be taken into consideration.

Four methods are used to calculate the coordinates of the transponders, including:

(1) The Least Squares solution with average sound speed is abbreviated as LS1. (2) The Least Squares solution with the cutoff angle (cutoff angle = 65°) is abbreviated as LS2. (3) The Least Squares solution without the average sound speed (cutoff angle = 65°) is abbreviated as LS3. (4) The new method for multiple transponders is abbreviated as SLS. 

**Figure 9:**
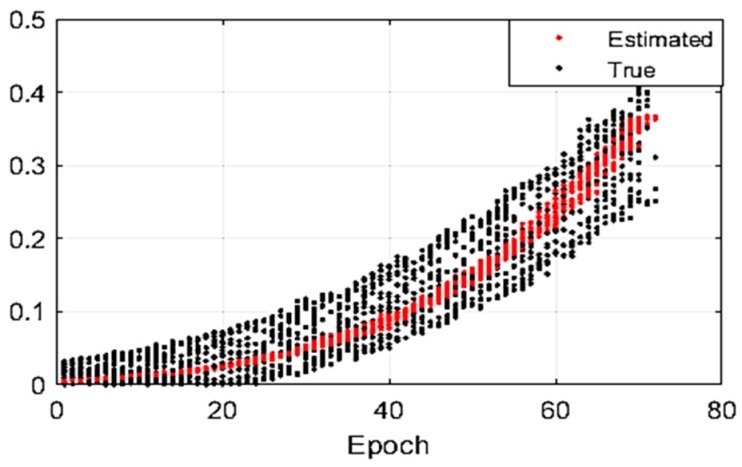
The correction of SRB by the SLS method and the value of SRB (depth: 100 m; month: January).

In particular, the SLS algorithm reordered the data according to the incidence angle for grouping calculation, so the epoch (Figure 9) does not have the significance of the time sequence. As shown in Figure 9, the SRB correction for 20 transponders can be calculated using the SLS method. The true SRB correction can reach up to 0.35 m at a depth of 100 m. Comparing the estimated correction with the true SRB, the fit validity of the SLS method for estimating SRB correction can be proved. The observation epochs of the 20 transponders are counted and the position bias of the three directions is calculated. The effectiveness of the new algorithm is also verified by the final positioning results. The mean position bias (MPB) is used to evaluate the accuracy of the different methods, and is defined by

(25) $$\mathrm{incidence}\mathrm{MPB}=\sum_{i=1}^{n}\mathrm{norm}({\left(\hat{x},\hat{y},\hat{z}\right)}_{i}-{\left(x,y,z\right)}_{i})/n$$

where $\left(\hat{x},\hat{y},\hat{z}\right)$ represent the estimated transponder coordinates and (x,y,z) are the true transponder coordinates. norm(.) represents modulus operation, n represents the number of transponders.

Table 1 shows the MPB of horizontal and vertical coordinates calculated using the above methods under good observation conditions. Each table includes three parts, corresponding to water depths of 100 m, 200 m and 300 m. As a result, even though the errors of SRB in the original ranging measurements are very large, the new method (SLS) is more effective at reducing the effect of the errors of SRB. We find that the new method can improve the results with centimeter-level accuracy in the horizontal and vertical components at depths of 100 m, 200 m and 300 m, respectively. The LS3 method can cancel the effect of SRB by estimating the sound speed, and the average positioning accuracy of transponders is higher than 10 cm in the horizontal direction. However, unlike the other methods, the new method is able to calculate the vertical components with centimeter-level accuracy as well, and the average accuracy of the vertical components is better than 4 cm. The actual observation conditions are worse than the above experiments; for example, the number of observations is insufficient and the observation structure is asymmetric. To investigate the effect of sampling period (good or bad observational conditions) on multiple transponders by different methods, the sampling rate is changed from 15 s to 45 s.

We find that the accuracy of the horizontal and vertical components tends to decrease with the decrease in the sample rate under bad conditions (see Table 2). In this case, the SRB correction is difficult to eliminate using a symmetrical structure, but the new SLS method can also improve the accuracy of the positioning. When the depth of the transponders reaches 300 m, the average accuracy of the horizontal components is better than 10 cm. 

For one transponder (depth = 300), the number of observations is about 1600/20 = 80, and most of the data have large incidence angles (see Figure 8b). To analyze the effect of observation structure on positioning accuracy, the mean position dilution of precision (MPDOP) (this is a measure of X, Y, Z position geometry) can be calculated by

(26) $$\mathrm{MPDOP}=\sum_{i=1}^{n}\sqrt{\left(q_{x_{i}}^{2}+q_{y_{i}}^{2}+q_{z_{i}}^{2}\right)}/n$$

where $q_{x_{i}}^{2}$, $q_{y_{i}}^{2}$ and $q_{z_{i}}^{2}$ are the elements of the covariance matrix of the estimated parameters, and n represents the number of transponders. The MPDOP (depth = 100 m, 200 m and 300 m) is as follows.

As shown in Table 3, LS1 is a more robust algorithm than the other three methods, and the MPDOP is minimal. The LS1 method is better than the LS2 method for taking full advantage of all the data, and the system is more stable. The stability of the SLS method is reduced with increasing model parameters. Although this may lead to a certain amount of instability in the solution, the actual positioning accuracy is improved with the more refined solution of SRB correction. 

### 4.2. Case 2: Experiment in the South China Sea

**Figure 10:**
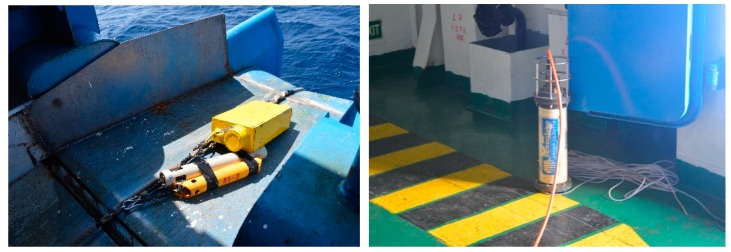
Installation and placement of the transponders (**left**) and transceiver (**right**). The transceiver consists of a rugged waterproof electronics bottle connected to a remote acoustic transducer, which was deployed. Lightweight acoustic transponder (Type 8044), operating frequency HF (35–50 kHz), dimensions (length × diameter) 490 mm (19.3”) × 63 mm (2.48”).

**Figure 11:**
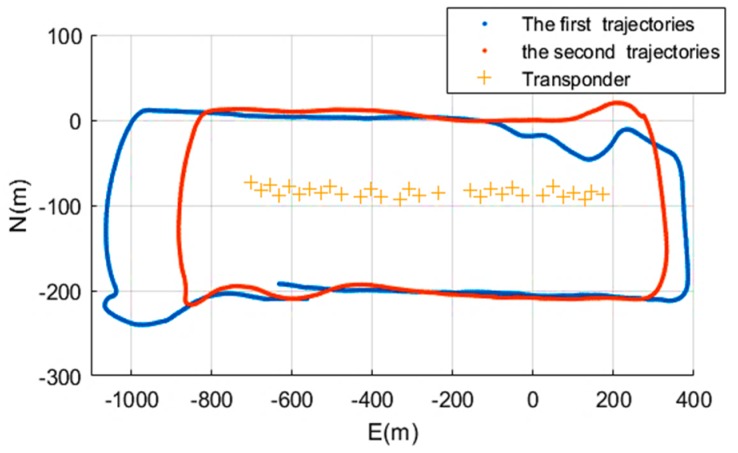
The trajectories of the experiment in the South China Sea. The blue line is the first surveying trajectory, and the red line is the second surveying trajectory.

As shown in Figure 10, the ship is equipped with GNSS, a transducer, a transponder SSP, and so on. The experiment area was on the east of Hainan Island in the South China Sea (Figure 11). The position of the ship was measured by a kinematic VeriPos DGNSS system. Once the transponders had been sunk in the sea, the exploration ship surveyed around them twice. The ship’s heading was recorded with an electric gyrocompass, and four GNSS antennas fixed around the ship were used to measure the ship’s roll and pitch [31], and the positioning of the transceiver on the bottom of the ship can be calculated using ship’s attitude data and DGNSS measurements. The mean sound speed measured by SSP is 1534 m/s. The water depth at the experiment site was approximately 100 m, and the acoustic ranging system included a transducer and 30 transponders. The acoustic positioning system included two types, an OBC acoustic positioning system developed by UK Sonardyne Company (Yateley, UK), and the China BPS acoustic positioning system; the sea trial was roughly 40 min. In this experiment, some BPS transponders had an electrical failure. Two trajectories were completed successively in the same day. For the first trail, the OBC acoustic system and the BPS acoustic positioning system were tested together. For the second trail, only the OBC acoustic system was tested.

**Figure 12:**
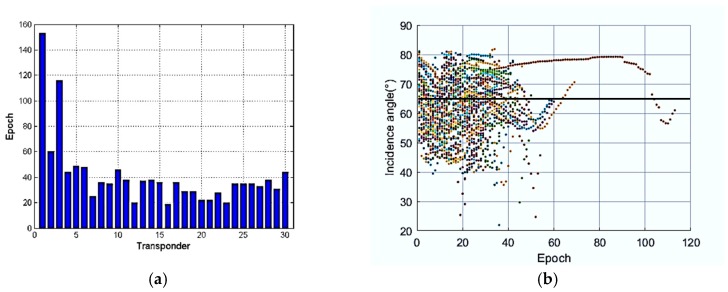
(**a**) The epoch number of the OBC transponders in the first experiment; and (**b**) incidence angles change with epoch of all OBC transponders.

The observation number of each transponder is shown in Figure 12a, and the average number of acoustic measurements of a transponder is about 40. No. 1 and No. 3 transponders responded so frequently because the ship began sailing with a time measurement near the first transponder for a long time. The dotted line of incidence angles with epochs is shown in Figure 12b. Nearly half of the observation data have an incidence angle greater than 65 degrees, and abandoning these observations could cause serious ill-posed problems and damage the positioning accuracy. As the number of observations of each transponder is not enough, acoustic observations with high incidence angle are necessary.

To estimate the absolute position of the seafloor transponders, we evaluate the positioning accuracy according to the mutual deviation of the two kinds of transponders. The positioning accuracy will increase, while mutual deviation decreases. Two different mean position biases (MPB1 and MPB2), using above approaches, are defined as

(27) $$\mathrm{MPB}1=\sum_{i=1}^{n}\mathrm{norm}({\left(\hat{x},\hat{y},\hat{z}\right)}_{\mathrm{OBC}}{}_{i}-{\left(\hat{x},\hat{y},\hat{z}\right)}_{\mathrm{BPS}}{}_{i})/n$$

(28) $$\mathrm{MPB}2=\sum_{i=1}^{n}\mathrm{norm}({\left(\hat{x},\hat{y},\hat{z}\right)}_{1i}-{\left(\hat{x},\hat{y},\hat{z}\right)}_{2i})/n$$

where $\left(\hat{x},\hat{y},\hat{z}\right)$ are the estimated transponder coordinates and (x,y,z) are the true transponder coordinates. MPB1 represents the first trail result using the OBC and BPS systems, and the MRMS2 is calculated using the OBC acoustic system only.

There are many outliers in the BPS observations, the BPS positioning results are not satisfactory, and only five transponders were chosen for investigation. Table 4 provides the absolute deviation of the four methods using the BPS acoustic system and the OBC acoustic system. Inaccurate sound speed measurements also affect the final positioning accuracy. The accuracy of the SLS is better than the other three methods, and the MPB1 in the horizontal direction is 0.54 m.

Two repeated observations of MPB2 on 30 transponders using the four methods can also show the performance of different algorithms. Table 5 provides the MPB2 of the LS2 and LS3 methods with different cut angles. As the cut angle increases, the positioning accuracy of the LS2 solution is improved, with the positioning accuracy reaching as high as 0.52 m and 0.64 m in the horizontal and vertical directions, respectively, when the cut angle was 75 degrees. Since a transponder has fewer observations, the accuracy of LS3 was lower than that of LS2, and the positioning accuracy was 0.64 m.

In Table 6, we have listed the horizontal and vertical MPB2 using the four methods. It can be seen that the SLS method can also improve the positioning accuracy more significantly than the other methods. The performance difference between SLS and the other methods is also clearly noticeable in favor of the SLS method, which can also be seen in Table 4. Experimental results show that the MPB2 of SLS method in the horizontal and vertical directions, respectively, are about 30 cm and 25 cm.

## 5. Conclusions

The SRB error is an important factor influencing underwater positioning. In this study, the effect of SRB has been taken into account, and we have constructed a new SIA model to estimate the SRB correction. The new method differs from the three conventional methods, and all acoustic data are fully utilized to estimate the parameters of the SRB model. Although high-incidence data will introduce SRB errors, these observations are highly conducive to the stability of the model. In the acoustic data procedure, we adopt a new method that yields beneficial results.

Simulation and experimental tests were carried out in order to evaluate the performance of the new method. The simulation results show that the average horizontal positioning accuracies of the new method at different depths are all better than 10 cm, even in the case of a poor positioning environment. Without being restricted to strict symmetrical observations, the other advantage of the new method is that it can effectively improve the accuracy in the vertical direction, and the vertical positioning accuracy can be substantially improved, from 0.8 m in the LS scheme to 0.03 m. The performance of the new method was also validated by experiments in the South China Sea, and the results show that the positioning accuracy can be substantially improved from 0.5 m in the LS scheme to 0.3, based on the SLS and SIA model. It is obvious that the novel method can perform better than the conventional single transponder positioning model, according to both simulation and experimental results. Further experiments and verification by means of deep sea data will be carried out in the future.

Further studies will include the following:

The stochastic model can be considered based on incidence angle or other factors (e.g., travel time and residual). When the observed data are insufficient, the equation can easily lead to ill-posed problems. The regularization or ridge estimation methods could be used to solve these problems.

## Figures and Tables

**Table 1:** The MPB and epoch by using the four methods under good conditions (m).

Method		LS1	LS2	LS3	SLS
**100 m**	**Epoch**	1865	791	791	1865
	**Horizontal**	0.06	0.09	0.09	0.06
	**Vertical**	0.10	0.04	0.09	0.03
**200 m**	**Epoch**	3024	1676	1676	3024
	**Horizontal**	0.10	0.06	0.06	0.05
	**Vertical**	0.48	0.04	0.12	0.03
**300 m**	**Epoch**	3200	2402	2402	3200
	**Horizontal**	0.57	0.05	0.05	0.050
	**Vertical**	0.51	0.05	0.26	0.028

**Table 2:** The MPB and epoch by using the four methods under bad conditions (m).

Method		LS1	LS2	LS3	SLS
**100 m**	**Epoch**	925	381	381	925
	**Horizontal**	0.13	0.16	0.16	0.09
	**Vertical**	0.13	0.09	0.16	0.08
**200 m**	**Epoch**	1297	612	612	1297
	**Horizontal**	0.15	0.15	0.10	0.06
	**Vertical**	0.50	0.12	0.12	0.04
**300 m**	**Epoch**	1536	777	777	1536
	**Horizontal**	0.15	0.09	0.09	0.08
	**Vertical**	0.89	0.16	0.23	0.03

**Table 3:** The MPDOP by using four methods in the good and bad condition (m).

Method		LS1	LS2	LS3	SLS
**100 m**	**Good**	0.41	0.43	0.64	0.43
	**Bad**	0.69	0.82	1.14	0.75
**200 m**	**Good**	0.34	0.37	0.47	0.35
	**Bad**	0.53	0.61	0.78	0.57
**300 m**	**Good**	0.30	0.33	0.45	0.32
	**Bad**	0.45	0.51	0.64	0.48

**Table 4:** The MPB1 of the positions with different cut angles using the four methods (m).

Method	LS1	LS2	LS3	SLS
**Horizontal**	0.66	0.97	0.56	0.54
**Vertical**	0.83	1.09	0.84	0.77

**Table 5:** The MPB2 of the positions with different incidence angles using the LS2 and LS3 methods.

	Incidence Angle/°	60	65	68	70	75	80
Method							
**LS2(m)**		0.77	0.66	0.60	0.52	0.53	0.52
**LS3(m)**		2.48	1.24	1.11	0.80	0.65	0.64

**Table 6:** The MPB2 of the positioning with four methods in horizontal and vertical direction (m).

Method	LS1	LS2	LS3	SLS
**Horizontal**	0.52	0.51	0.56	0.30
**Vertical**	0.36	0.42	0.96	0.25
